# Flow Diverter in Unruptured Intracranial Vertebral Artery Dissecting Aneurysm

**DOI:** 10.3389/fneur.2022.912863

**Published:** 2022-06-21

**Authors:** Han San Oh, Jin Woo Bae, Chang-eui Hong, Kang Min Kim, Dong Hyun Yoo, Hyun-Seung Kang, Young Dae Cho

**Affiliations:** ^1^Department of Neurosurgery, Seoul National University Hospital, Seoul National University College of Medicine, Seoul, South Korea; ^2^Department of Neurosurgery, Inha University Hospital, Incheon, South Korea; ^3^Department of Neurosurgery, Veterans Health Service Medical Center, Seoul, South Korea; ^4^Department of Radiology, Seoul National University Hospital, Seoul National University College of Medicine, Seoul, South Korea

**Keywords:** aneurysm, vertebral artery, dissection, reconstructive, flow diverter

## Abstract

**Objective:**

Intracranial vertebral artery dissecting aneurysm (VADA) may present as aneurysmal dilation alone, dilation with coexisting stenosis, or, in some cases, as a recurrent aneurysm after previous reconstructive treatment. To date, the clinical utility of flow diverters in VADA has not been examined according to these various circumstances. This study aims to report the safety and efficacy of flow diverters in the treatment of various manifestations of intracranial VADA.

**Methods:**

A total of 26 patients and 27 VADAs treated with flow diverting stents from November 2014 to September 2021 were included. Medical records and radiologic data were analyzed to assess the safety and efficacy of flow diverting stents.

**Results:**

The results showed that 12 cases (44.4%) presented with aneurysmal dilation only, 7 (26.0%) with aneurysmal dilation and one or more associated stenotic lesions, and 8 (29.6%) as recurrence after previous treatment, including stent-assisted coil embolization (*n* = 5), single stent only (*n* = 1), and coil embolization without stent (*n* = 2). Among 27 lesions, 25 were treated with single flow diverters; additional flow diverting stents were required in 2 cases because of incomplete coverage of the aneurysm neck. There was one instance of incomplete expansion of the flow diverter. All cases showed contrast stagnation in the aneurysmal sac immediately after deployment of the flow diverting stent, and during a mean follow-up period of 18.6 months (range 6 to 60), the overall complete occlusion rate was 55.6%, with complete occlusion of 83.3% of aneurysmal dilation only lesions, 42.9% of aneurysms with stenosis, and 25% of the recurrent aneurysm. Only two patients (7.7%) had delayed ischemic complications.

**Conclusion:**

Flow diverters have proven safe and effective in unruptured VADA. However, the complete occlusion rate with the flow diverter is relatively lower in VADA with stenosis or with previous stent placement than in dilation-only lesions. Further study with a larger cohort would be needed to confirm these results.

## Introduction

Even though vertebral artery dissection (VAD) is known as a rare disease, it is associated with potentially critical cerebrovascular lesions with a significant variety of unspecific symptoms (1). VAD is a dynamic process that is variably manifested at angiography as stenosis, occlusion, and aneurysmal dilation, and clinical presentation of VAD is also diverse (e.g., asymptomatic, headache, neck pain, neurologic mass effect, posterior circulation stroke, or subarachnoid hemorrhage). Although the treatment of ruptured VAD is relatively well established due to its protection against rebleeding, the justification and modalities for the treatment of unruptured VAD are still controversial.

The reported incidence of the aneurysm, referred to here as vertebral artery dissecting aneurysm (VADA), is as high as 64.9% in patients with unruptured intracranial VAD (2). Intracranial VADA is associated with various circumstances, including aneurysmal dilation with or without stenosis and recurrent aneurysm, after reconstructive treatment. The majority of asymptomatic VADAs are resolved with conservative treatment, but rupture is related to high mortality and morbidity (3–6) and a large VADA (>10 mm) may signal a worse clinical course (7, 8). Therefore, treatment is indicated for symptomatic aneurysms or those larger than 10 mm (7, 9). With advances in endovascular technologies, the use of flow diverters in the reconstruction of VADAs is increasing. However, to date, there is still no literature comparing treatment outcomes for flow diverters according to the various clinical circumstances. Our study aimed to evaluate the safety and efficacy of flow diverting stents in the treatment of intracranial VADA in cases of aneurysmal dilation with and without accompanying stenosis and in cases of recurrent aneurysm.

## Materials and Methods

### Patient Population

From November 2014 to September 2021, a total of 90 intracranial VADAs were treated with endovascular treatment at our institution. Of these, flow diverting stents were used in 31 unruptured aneurysms (34.4%), and clinical and radiologic follow-up evaluations were available for all patients (26 patients with 27 aneurysms) at least 6 months later, except for four recently treated.

In our institution, conservative treatment was preferred in unruptured VADAs. Treatment was considered for aneurysms that expanded in size during follow-up examinations, became symptomatic producing headache and neck pain, or displayed mass effect or recurrent ischemic events. Asymptomatic patients who feared rupture and young or middle-aged patients with large-sized VADAs (> 10 mm) underwent reconstructive endovascular treatment after appropriate discussion. And, additional treatment was indicated for recurrent aneurysms after prior treatment with reconstructive modalities, such as stent-assisted coiling. Aneurysms occurring by concurrent major trauma, angiitis, or vasculopathy were excluded.

Therapeutic decisions (including flow diverter, multiple stenting, or stent-assisted coil embolization) were determined for each patient after considering treatment benefits, risks, and alternatives (including medical treatment and surgical clipping), through multidisciplinary deliberation of both neurosurgeons and non-surgical neurointerventionists, and informed consent was obtained from all patients.

This study received approval from our Institutional Review Board (No. SNUH 2201-061-1290) and required patient consent for this retrospective investigation was waived.

### Preparation for Endovascular Procedures

In each instance, procedures were performed under general anesthesia, administering antiplatelet medication beforehand. Our institutional protocol calls for dual antiplatelet agent use (prasugrel and aspirin). All patients with unruptured VADA routinely received loading doses of prasugrel (20 mg) and aspirin (300 mg), given the days before a procedure, supplemented by prasugrel (5 mg) and aspirin (100 mg) the next morning. During procedures, a bolus of heparin (3,000 IU) was given upon placement of the femoral arterial sheath and thereafter sustained by hourly doses (1,000 IU) and the activated clotting time was monitored each hour.

### Endovascular Procedure and Outcome Evaluation

All procedures were performed using Allura Clarity (Philips Medical Systems, Best, Netherlands). Under general anesthesia, using the Seldinger technique, the right side common femoral artery was punctured. After the introduction of a 6-Fr guiding sheath (Flexor Shuttle, Cook Medical) into the aortic arch, a 3,000 IU intravenous heparin bolus was injected peripherally to maintain an activated clotting time between the 250 s and 300 s. An intermediate catheter (5 or 6-Fr) was inserted into the lesioned VA and rotational angiography was performed to obtain the working projection. Then, under the guidance of a “0.014 microwire, the 0.027” microcatheter was advanced into the VADA.

The most frequently used diverters in our cohort were the Medtronic pipeline embolization device (PED, *n* = 16 cases), the Stryker Neurovascular Surpass Evolve (*n* = 9), and the Microvention flow-redirection endoluminal device (FRED, *n* = 2). All flow diverting stents were deployed from the normal parent artery distally to the normal parent artery proximally to cover the entire dissecting segment. It was intended that the distal marker of the flow diverting stent not reach the vertebrobasilar junction. Wall apposition status was evaluated on a control angiogram, and if better wall apposition was needed, angioplasty using an ultra-compliant balloon was performed. For postoperative management, dual antiplatelet agents (prasugrel [5 mg] and aspirin [100 mg]) were maintained for 1 month, after which prasugrel was replaced by clopidogrel (75 mg). The modified dual antiplatelet medication regime was maintained for 3 or 5 months (after June 2020) in all patients, after which a single antiplatelet agent was maintained.

### Clinical and Radiologic Follow-Up Evaluation

For follow-up radiologic evaluation, digital subtraction angiography (DSA) was taken at 6 months as a routine, and magnetic resonance angiography (MRA) was performed serially at 18 and 36 months. Additional DSA was taken 12 months later if complete occlusion was not observed at the 6smonths follow-up. During the follow-up period, the radiologic outcome was classified using the Raymond classification (10): [Class 1, complete occlusion (no contrast filling at the aneurysm site); Class 2, residual neck (contrast stagnation at the base of the aneurysm); Class 3, residual aneurysm (contrast stagnation at aneurysm sac)]. The remnant neck and residual sac were described with the O'Kelly-Marotta (OKM) grading scale (11). The radiologic assessment was performed by two qualified neurointerventionists.

Clinical outcome was evaluated by the modified Rankin Scale (mRS), which was recorded at the point of initial hospital admission and the last hospital visit.

## Result

### Baseline Patient Characteristics

The total number of aneurysms was 27 (14 men and 13 women). The mean age of the patients was 55.2 (37 to 75) years. Except for one patient with bilateral aneurysms, all patients had single aneurysms. There were 16 aneurysms with acute symptoms, including ischemic stroke (*n* = 6, mRS 1 in 3, mRS 2 in 2, and mRS 3 in 1), headache (*n* = 4, all mRS 1), neck pain (*n* = 1, mRS 1), or neurologic mass effect (*n* = 4, mRS 1–4 distributed in one each). Twelve aneurysms (in 11 patients) were asymptomatic, but all were lesions larger than 10 mm. Eight of them had an increase in the size of DA during follow-up (mRS 3 in 1 due to previous SAH).

The mean maximal length of the dissecting aneurysm was 15.4 mm (range from 6.8 to 27.3 mm). Among the aneurysms, 12 (44.4%) showed aneurysmal dilation only, 7 (25.9%) showed aneurysmal dilation with stenosis, and 8 (29.6%) were recurrent aneurysms after previous treatment (five by stent-assisted coil embolization, one with sole stent, and two by coil embolization without stent). Six aneurysms (23.1%) had an incorporated branch, either the anterior spinal artery (ASA; 1 case) or the posterior inferior cerebellar artery (PICA; 5 cases). All patient characteristics are summarized in Table 1.

**Table 1:** Clinical and radiologic characteristics of subjects depending on the various circumstances.

	**Total (27)** **No. (%)**	**Aneurysm dilatation only (12)**	**Aneurysm dilatation with stenosis (7)**	**Previous treatment**	
				**SACE + Sole stent (6)**	**CE (2)**
**Clinical factors**					
Sex, male	14 (51.9)	8	3	3	0
Age (range)	55.2 (37–75)	53.4 (41–71)	57.9 (45–70)	51.7 (37–66)	67.5 (60–75)
Hypertension	18 (66.7)	6	5	5	2
Diabetes	5 (18.5)	2	0	2	1
Hyperlipidemia	13 (48.1)	6	5	2	0
Coronary artery disease	3(11.1)	2	0	1	0
Smoking	7 (25.9)	3	2	2	0
**Clinical presentation**					
Neurologic mass effect	4(14.8)	1	0	2	1
Ischemia	6 (22.2)	2	3	1	0
Headache	4 (14.8)	0	2	2	0
Neck pain	1 (3.7)	1	0	0	0
Asymptomatic	12 (44.5)	3	1	0	0
Increased aneurysm size	8	5	1	1	1
**Aneurysm factors**					
Dissection length (range)	15.4 (6.8–27.3)	13.3 (6.8–17.9)	17.4 (7.6–27.3)	17.6 (10–25)	13.8 (10.5–17.1)
Aneurysm depth (range)	9.2 (4.1–23.6)	8.1 (4.2–11.1)	6.8 (4.1–8.8)	13.3 (7.6–23.6)	13.0 (5.97–19.9)
**Aneurysm type**
Circumferential	22 (81.5)	10	6	4	2
Eccentric	5 (18.5)	2	1	2	0
Aneurysmal dilation with stenosis	11(40.7)	0	7	3	1
PICA involvement	5 (18.5)	2	3	0	0
ASA involvement	1 (3.7)	0	0	0	1
Dominant vertebral artery	14 (51.9)	5	5	3	1
Aneurysmal thrombus	11 (40.7)	4	2	4	1
Acute angulation of stented artery	5(18.5)	1	3	0	1
No of angulation in stented artery
= 1	22	10	5	6	1
≥ 2	5	2	2	0	1

*SACE, Stent-assisted coil embolization; CE, Coil embolization; PICA, Posterior inferior cerebellar artery; ASA, Anterior spinal artery; No, number*.

### Procedure Outcome

The single stent was used in 25 cases. Additional flow diverting stents were required in 2 cases because of incomplete coverage of the neck of the aneurysm (stent shortening due to stent expansion into the sac), and incomplete expansion of the stent occurred in one patient because of arterial tortuosity. In the latter, passage the balloon to expand the stent failed despite all efforts. All three patients above were clinically asymptomatic.

All cases showed contrast stagnation in the aneurysmal sac immediately after placement of the flow diverting stent. There was no periprocedural complication, but the delayed complication rate was 7.4% (2/27; 1 transient ischemic attack and 1 lateral medullary infarction).

### Clinical and Radiological Follow-Up Outcomes

**Figure 1:**
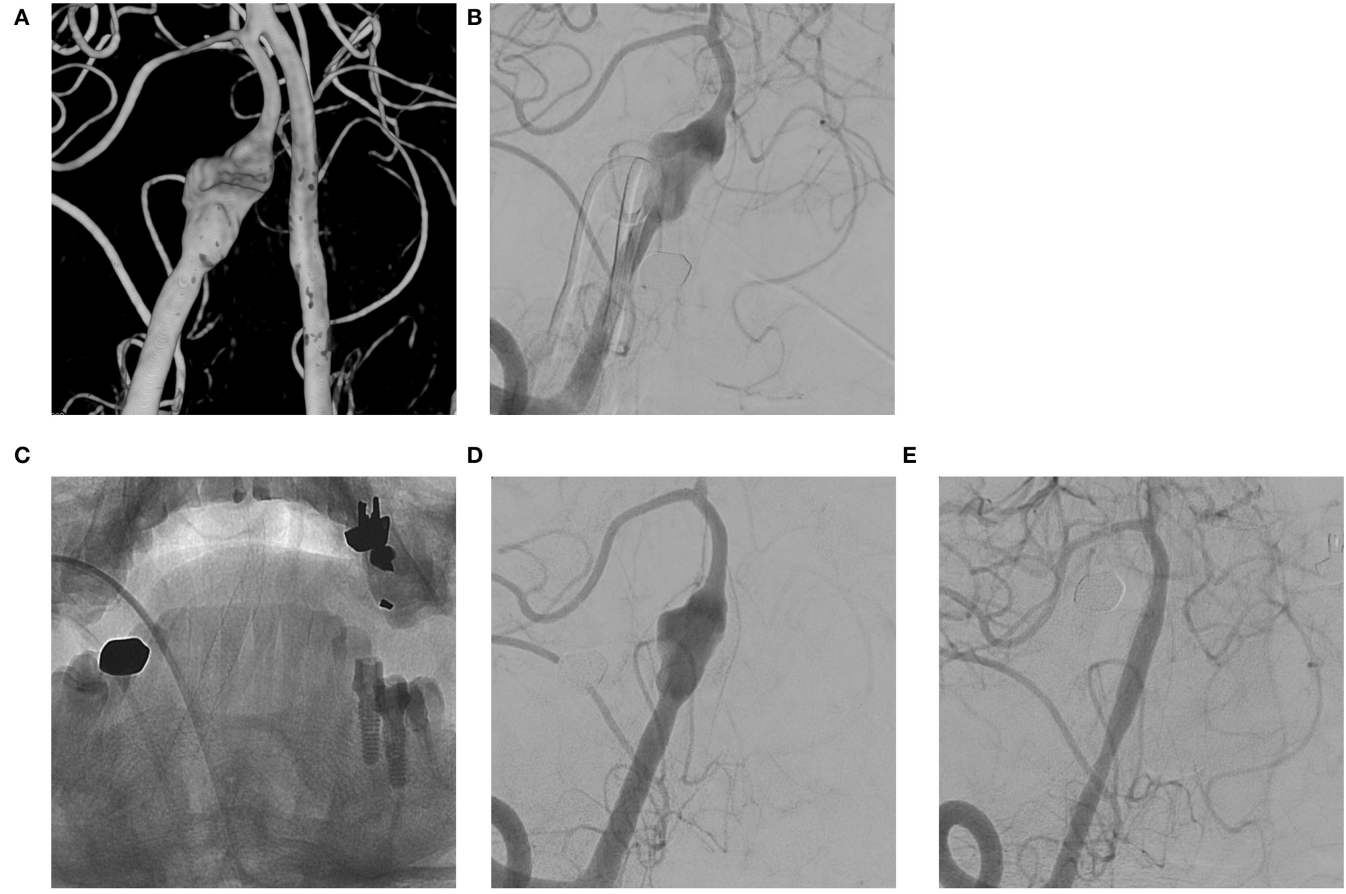
**(A,B)** VADA with dilation only without stenosis at the right distal vertebral artery; **(C,D)** Flow diverting stent deployed at the lesion; **(E)** Complete occlusion of the aneurysm at 6 month follow-up angiography.

**Figure 2:**
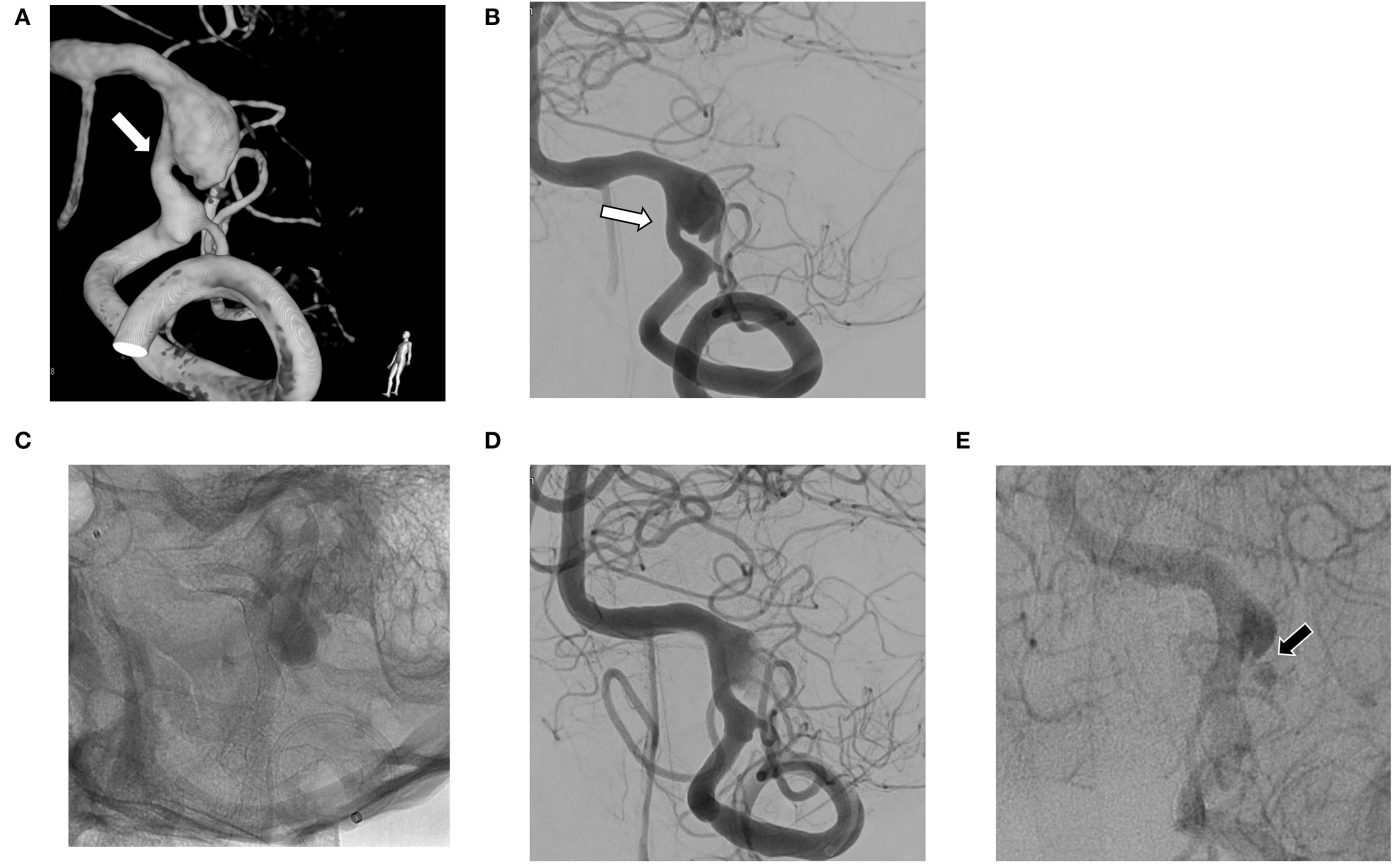
**(A,B)** Vertebral artery dissecting aneurysm (VADA) combined with fusiform dilation and stenosis (white arrow); **(C,D)** The flow diverting stent deployed at the VADA fully covers the lesion; **(E)** Small residual neck and delayed sac filling (black arrow) at 6 month follow-up angiography.

**Figure 3:**
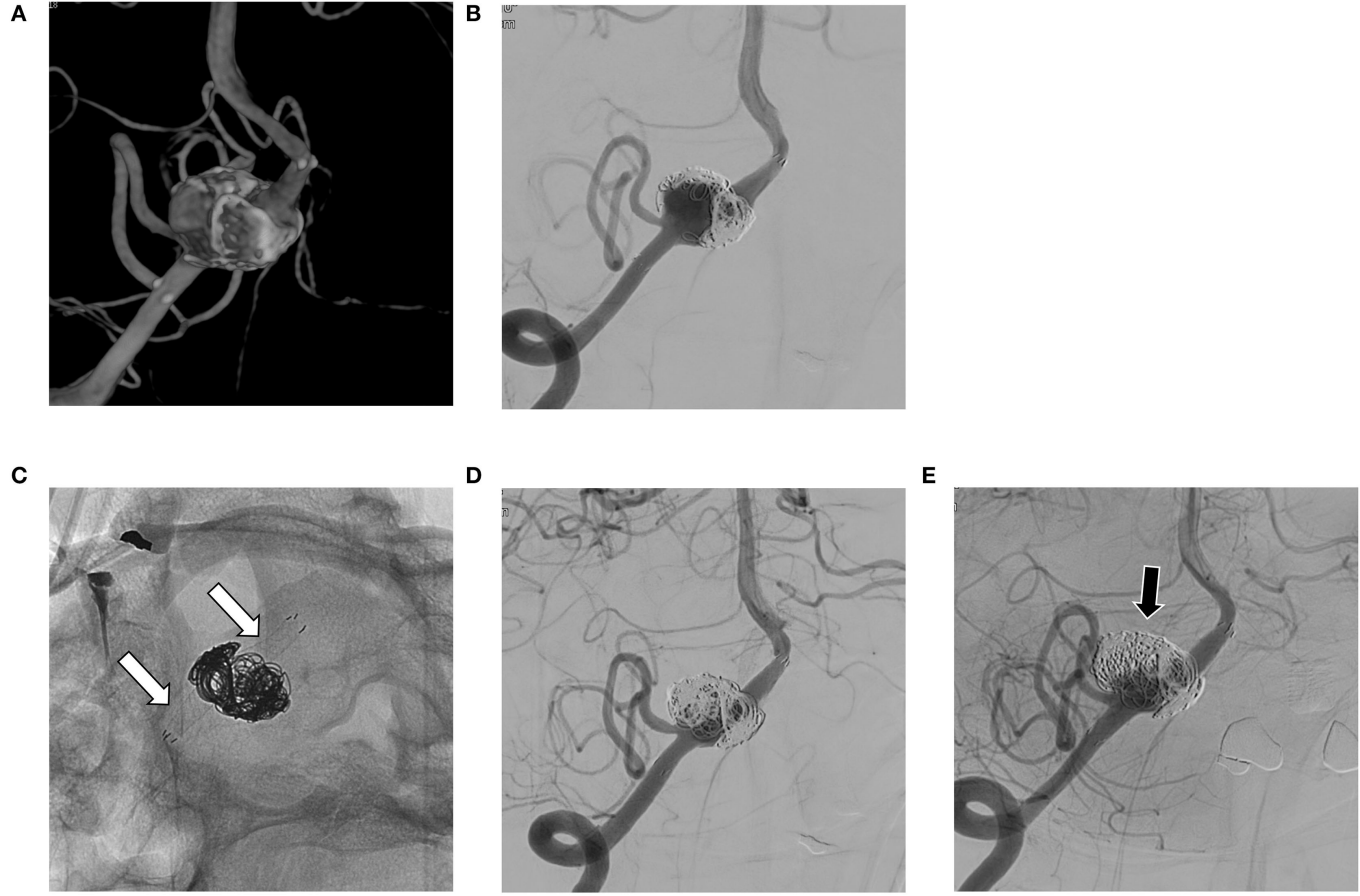
**(A,B)** Recurrent aneurysm is noted at 6 months after stent-assisted coil embolization in the right vertebral artery; **(C,D)** A flow diverting stent is deployed over the previous stent (white arrows in C); **(E)** On follow-up angiography after 24 months, the residual sac remains (black arrow).

The mean follow-up period was 18.6 months (range, 6 to 60). The complete occlusion rate at 6 months was 51.9% (14/27). The rate of residual sac and remnant neck at 6 months was 37% (10/27) and 11.1% (3/27), respectively. Three of the residual sacs were OKM grade A2, another 3 were B2, and the other 4 were B3; among the remnant necks, 2 were OKM grade C3 and 1 was OKM grade C2. Overall, the complete occlusion rate after 6 months was 55.6% (15/27). The complete occlusion rate for lesions with only aneurysmal dilation during 6 to 60 months was 83.3% (10/12) (Figure 1), whereas the complete occlusion rate of other aneurysms (those with stenosis or previous treatment history) during 6 to 48 months was 33.3% (5/15). Among aneurysms with stenosis, the complete occlusion rate during 6 to 18 months was 42.9% (3/7) (Figure 2), and among recurrent aneurysms, the occlusion rate during 18 to 48 months was 25% (2/8) (Figure 3). The details are summarized in Table 2.

**Table 2:** Radiologic and clinical follow-up outcome after flow-diverter.

	**Total (27)** **No. (%)**	**Aneurysm dilatation only (12)**	**Aneurysm dilatation with stenosis (7)**	**Previous treatment**	
				**SACE + Sole stent (6)**	**CE (2)**
**6 month follow-up**					
Complete occlusion	14 (51.9)	9	3	2	0
Residual neck	3 (11.1)	2	1	0	0
Residual sac	10 (37.0)	1	3	4	2
**Cumulative follow-up, mo**	18.6 (6–60)	16 (6–60)	10.3 (6–18)	32.7 (18–48)	21 (6–36)
Complete occlusion	15 (55.6)	10	3	2	0
Residual neck	5 (18.5)	2	1	1	1
Residual sac	7 (25.9)	0	3	3	1
**Procedural event**					
Incomplete neck coverage	2 (7.4)	2	0	0	0
Incomplete stent expansion	1 (3.7)	0	0	1	0
**Delayed complication**					
Ischemic complication	2 (7.4)	1	0	0	1
**Clinical outcome (mRS)**
Improved mRS	11 (40.7)	6	1	3	1
Worsened mRS	1 (3.7)	1	0	0	0
NIC of mRS	15 (55.6)	5	5	4	1

*SACE, Stent-assisted coil embolization; CE, Coil embolization; mRS, modified Rankin scale; NIC, No interval change; mo, month*.

Most of the patients (*n* = 25, 96.2%) had a good clinical outcome (mRS ≤ 2). Of the four patients who showed clinical symptoms due to mass effect, three showed improvement in mRS and one showed no interval change. One patient had deterioration of mRS after flow diversion because of delayed ischemia from a lateral medullary infarction despite complete occlusion of the aneurysm.

## Discussion

Though VADA is a less common intracranial aneurysm, once it ruptures, it is associated with high morbidity and mortality (3–6). Symptomatic VADA presents with ischemia and hemorrhage. In particular, large VADA, when compared with small or steno-occlusive VAD, has exhibited a worse clinical course (7, 8). Therefore, for lesions larger than 10 mm, treatment is indicated when there is evidence of size increase or development of symptoms related to the involved vertebral artery (9, 12). Intracranial VADA may present as aneurysmal dilation with or without stenosis or as a recurrent aneurysm following prior reconstructive treatment, such as stent-assisted coiling, coiling alone or stent alone, or other. Individualized strategies should be considered in treating these various aneurysms, but the evidence is lacking.

Surgical treatment for VADA has been associated with high morbidity and mortality (13), and endovascular treatment has been the preferred primary intervention (14). Endovascular methods are described as deconstructive, i.e., parent artery occlusion, and reconstructive, i.e., stent-assisted coil embolization and flow diverting stenting. Parent artery occlusion has been deemed effective and safe, but if the aneurysm is located in the dominant VA or incorporates important branches, such as the PICA or ASA, occlusion of the parent artery cannot be an option because of the risk of ischemic complications, including cerebellar, medullary, and spinal cord infarction (15, 16).

Therefore, our institution has preferred reconstructive endovascular treatment in unruptured VADA. Stent-assisted coil embolization has the advantage of immediate occlusion. On the other hand, it has been associated with recurrence in the case of incorporated branches or incomplete coil packing in the aneurysmal sac. For VADA incorporating the PICA with circumferential shape, the technical difficulty would be expected (17), and for these cases, a flow diverting stent can be a good alternative.

The literature (18–20) has reported complete occlusion rates of intracranial VADA with flow diverting stent of 31 to 75% and complication rates of 6 to 17%. To our knowledge, our case series has the largest patient population to date, and our results are comparable to the previous results. We also evaluated the details of cases of coexisting stenosis and cases of recurrent aneurysms. VADA with dilation only (see Figure 1) treated with flow diverting stents showed excellent results. This may be because the VA where the lesion is located has a relatively simple vascular path, so the flow diverting stent adheres well to the vascular wall. On the other hand, in VADA with stenosis, flow diverters were less effective at achieving complete occlusion. This may be due to the poor attachment of the flow diverting stent to the lesioned arterial wall. This phenomenon is also observed with recurrent VADA with the additional placement of a flow diverter within an already deployed stent (see Figure 3).

Several cascading mechanisms work for flow diverting stents to heal aneurysms; decreasing aneurysmal inflow, antegrade flow diversion at the lesion artery, generating thrombus formation, and luminal healing along the stent by epithelialization (21–23). Wall apposition is known to have a key role in aneurysm occlusion (24), and stenosis seemed to interrupt good wall apposition, causing endoleak (25). A previous stent might also interrupt wall apposition. The reported rates of complete occlusion in cases of recurrent aneurysms after treatment with stent assist coil embolization range from 38 to 65%, and compared with non-stent aneurysms, the treatment outcome of those aneurysms showed less efficacy of flow diverting stents (26–29). In summary, poor vessel wall apposition of the flow diverter leads to a reduced hemodynamic effect. Therefore, caution is required when applying a flow diverting stent in VADA with stenosis or with a previously deployed stent.

During follow-up of patients treated with flow diverting stent, ischemic event, occurring in approximately 5% of cases, is a well-known complication (30). There were two cases (7.7%) of ischemic complications in our series. One patient had transient ischemia, and the other one suffered delayed lateral medullary infarction with remaining neurologic morbidity (mRS 3). The patient had bleeding gums 3 months after the flow diverting stent. Therefore, the dual antiplatelet agent was modified to a single antiplatelet agent (75 mg of clopidogrel). The infarction occurred 2 months after the medication change. According to the flow diverter results in a ruptured VADA reported by Maybaum et al. (1), hemorrhagic complications and ischemic complications occurred in 9.6%, respectively.

Our investigation is limited in that it is a non-randomized retrospective observational study with a small study population. Therefore, meaningful statistical analysis could not be performed because the number of patients was small. Further large-cohort studies would be necessary to confirm our results. Also, the follow-up period was not uniform, and in some cases, the follow-up period might not have been sufficient to observe the flow diversion. In addition, since post-embolization diffusion-weighted MRI was not performed in all cases, asymptomatic ischemic events might have been excluded.

## Conclusion

In unruptured VADA, a flow diverter may be a safe and effective tool. However, in VADA with stenosis or previously treated with a stent, the complete occlusion rate with a flow diverter is relatively lower than in dilation-only lesions. Further studies with larger cohorts are required to confirm these results.

## Data Availability Statement

The original contributions presented in the study are included in the article/supplementary material, further inquiries can be directed to the corresponding author/s.

## Ethics Statement

The studies involving human participants were reviewed and approved by Seoul National University Hospital. Written informed consent for participation was not required for this study in accordance with the National Legislation and the Institutional Requirements.

## Author Contributions

HSO, YDC, and H-SK designed the manuscript and drafted the final version. YDC, DHY, KMK, and H-SK performed the interventions. HSO, JWB, and C-eH were responsible for data curation and statistical analysis. HSO and YDC designed the figures. All authors contributed to the article and approved the submitted version.

## Conflict of Interest

The authors declare that the research was conducted in the absence of any commercial or financial relationships that could be construed as a potential conflict of interest.

## Publisher's Note

All claims expressed in this article are solely those of the authors and do not necessarily represent those of their affiliated organizations, or those of the publisher, the editors and the reviewers. Any product that may be evaluated in this article, or claim that may be made by its manufacturer, is not guaranteed or endorsed by the publisher.
